# Alveolo-Dental Abscess

**Published:** 1879-01

**Authors:** D. D. Smith

**Affiliations:** Professor of Mechanical Dentistry, Philadelphia Dental College.


### ARTICLE III.

### *Alveolo-Dental Abscess.*

BY D. D. SMITH, M. D., D. D. S.,

*Professor of Mechanical Dentistry, Philadelphia Dental College.*

(Read before the Odontographic Society of Pennsylvania, Nov. 6th, 1878.)

Alveolo-dental abscess is a common surgical affection, often attended with extreme suffering, and more or less serious consequences, according to the condition of the patient, the structure of the alveolar tissue concerned, and the location of the tooth. It is usually amenable to rational treatment, admitting of cure without loss of the tooth. It may be defined as an abnormal cavity containing pus, having its seat in the cancellated structure between the external and internal aveolar plates,—the result of degenerative changes in the root, pulp, peridentium, or associate parts.

A sac is generally formed through an effusion of lymph, limiting the pus-forming surface, and accommodating itself to the quantity of broken-down tissue present. This sac or limiting membrane is commonly situated immediately above or below the root of a tooth (according as it is in the upper or lower jaw) with which it communicates through the apical foramen. In single-rooted teeth it is nearly always in this relation, but in double or multi-rooted teeth it is not infrequently found between the roots, and occasionally just at the bifurcation.

Should a root causing an abscess pierce the antrum of Highmore, as is sometimes the case with the second superior bicuspid and the first and second superior molars, the sac may form in this cavity, giving rise to antral troubles.

Alveolo-dental abscesses are of two kinds, viz., acute and chronic. The acute variety exhibits the same symptoms as an acute abscess in other parts,—violent throbbing pain, redness, heat, swelling, tension, altered circulation, and the formation of new products ; with a tendency to open by ulcerative absorption on the nearest surface.

Chronic alveolo-dental abscess may result from change in character or long continuance of the acute, or may exhibit subacute symptoms from the beginning. It is lacking in the activity and violent manifestations of the acute. The pain, while constant, is rarely intense ; the discoloration is not marked ; the temperature is but little increased ; the swelling is usually but slight, and the tendency to new products not decided in character.

A marked and even diagnostic symptom is, inclination to infiltration of pus into the surrounding tissues. It may excavate the alveolar structure, involving two or more adjoining teeth, or it may find its way through the alveolar plate and burrow in the submucous tissue, dissecting its way between the periosteum and the bone (as the palate processes of the superior maxillæ,) ultimately causing necrosis.

Such manifestations are found in persons of a strumous diathesis, and in the weak and debilitated from whatever cause.

A chronic alveolo-dental abscess, following an acute, is usually in connection with neglected roots, but may be associated with any tooth where the cause is allowed to remain.

The duration of the acute may be stated as from eighteen hours to ten days. With the exit of pus through the osseous structures the active manifestations, except the swelling, subside, and becoming chronic the discharge is continued, at intervals or continuously, as long as the cause remains; this may be for weeks, months, or even years.

Anæmia, strumous diathesis, the lymphatic temperament, pregnancy, and intemperance may be considered as predisposing causes. The exciting causes approximatively in the order of frequency are putrescent pulps, crownless roots of teeth, necrosis, pivoted roots (the canals containing putrescent pulps,) filling materials which have been forced through the apical foramen, salivary calculus, and mechanical violence to a tooth, as a blow.

An alveolo-dental abscess may point and open in one of nine ways: 1st, through the external alveolar plate and gums directly into the mouth; 2d, through the internal alveolar plate and gums directly into the mouth; 3d, externally or internally through the alveolar plates, emerging by a sinus through the mucous membrane at a point remote from the seat of the trouble; usually near the tuberosity of the superior maxilla, or behind the uvula; 4th, through the apical foramen and canal of the root, the pus escaping into the mouth from the crown of the tooth; 5th, from the seat of the abscess, downwards if in the upper jaw, upwards if in the lower jaw, between the peridentium and the cementum, and appearing at the free margin of the gum; 6th (in the lower jaw,) from the under surface of the *body* of the bone, and thence directly through the integuments; 7th (in the upper jaw,) externally through the cheek; 8th, into the antrum of Highmore, the pus passing through the middle meatus, to find exit from the nostril on the side of the lesion; 9th (when the trouble is in connection with the lower dentes sapientiæ, situated far back in the coronoid process or at the junction of that process and the body,) downwards and inwards through the osseous structures, under the cervical fascia, and thence to the thorax.

The prognosis of an abscess, in connection with single-rooted teeth, is almost always favorable.

(In classes fifth and ninth tetanus has sometimes supervened, and death from pyæmia has occasionally resulted from cases described in class fifth.)

The prognosis as regards the restoration of the tooth is favorable, excepting *lower bicuspids in class fifth*, and teeth described in classes sixth and ninth.

The more common sequelæ of alveolo-dental abscesses are, continuous discharge of pus; thickened peridental membrane causing a prolonged condition of "sore tooth;" facial neuralgia; indurations; osseous tumors, especially on the body of the lower jaw; necrosis of some portion of the maxillæ; trismus, and pyæmia.

The diagnosis is to be made from both general symptoms and the local manifestations.

In the acute form the general symptoms are, fever with its usual accompaniments of hot, dry skin, coated tongue, constipated bowels; more or less prostration, and often violent pain shooting down the neck or up through the face and head.

The local manifestations are more decided. The tooth at first seems elongated, and is "sore" to the touch; a dull pain begins in the socket, but increases in intensity, and soon becomes throbbing; the parts in the immediate vicinity are more and more responsive to pressure, swelling and discoloration following. These phenomena continue and augment so long as the pus is confined within the alveolar walls, but coincident with its escape into soft tissue, the inflammatory activities are quickly modified, save only the swelling, which increases until the evacuation of the pus externally.

The treatment of alveolo dental abscess may be either palliative or curative. Palliative treatment may be both general and local. The former consists in the use of morphia, opium, assafœtida, or quinia, according to the indications of the case; the latter is the more important, except in cases of great severity. It may consist in the application of extract of hamamelis, tincture of arnica or aconite, aconitia ointment (1 grain to 1 drachm of cerate,) poultices, or the use of pepper-bags to induce free suppuration, and to prevent cacoplastic deposits.

Early extraction should be resorted to in all cases of crownless roots (excepting such as are valuable,) partly erupted lower dentes sapientiæ, and most cases of bicuspids or molars which have no antagonists,—especially if there be considerable recession of the gum and alveolar structure.

A recent alveolo-dental abscess with a fistula opening directly through the gum, particularly if associated with single-rooted teeth, responds promptly and satisfactorily to well-directed treatment. If the abscess be in connection with a small and tortuous canal, as that often found in the anterior buccal root of a superior molar, or the compressed anterior root of a lower molar, the treatment is complicated by the mechanical difficulty of gaining access to the seat of the trouble.

In these, as in other cases, putrescent matter and *debris* of whatever kind should be thoroughly removed from the canal, and the entire pulp-cavity disinfected.

The cleansing of canals is best done by first protecting them from saliva, and wiping the cavity of decay dry; then with the free use of alcohol, a little cotton, and a probe of such shape as will readily pass into the canal, the offending matters may be easily and satisfactorily removed.

If then, with a probe wound with cotton, a little oil of cloves or creasote (preferably the latter) can be forced through the apical foramen, and thence out at the external fistula, the cure will be materially hastened; but drilling through the root to effect this should never be resorted to. (A white eschar at the mouth of the fistula indicates the passage of the creasote.)

Whether the medicament passes the apical foramen or not, it is better to dress the root with medicated cotton until the fistula is satisfactorily healed.

To dress a root, a few fibers of cotton are twisted into a thread one to two inches long, of a size to pass easily into the canal; the small end being dipped into the medicament (glycerine, creasote, or oil of cloves,) the thread is passed to the end of the canal, and allowed to remain there. In whatever way the crown-cavity may be temporarily stopped, the root dresеings should be easy of access. If the work of cleansing the canals has been thoroughly done, but two or three of these cotton dressings, renewed at intervals of two to four days, will usually be required to effect a cure.

If the pus, instead of establishing an opening through the gum, has found exit through the canal, the treatment is more complicated and much less certain in its results. As before, the pulp-cavity and canal should be carefully cleansed and disinfected, and the medicated cotton dressings (preferably oil of cloves or glycerine) applied. The cotton, however, should in such cases be so arranged as to be at the full command of the patient, to whom instructions should be given for its immediate removal on experiencing a sensation of *fullness* in the tooth,—this being the premonitory symptoms of renewed inflammation in the peridentium. Under no circumstances, in these cases, should the first cotton be allowed to remain more than three or four hours. It is better that it should be removed by the operator than by the patient. Absorbent cotton, unmedicated, introduced and kept dry, is a valuable adjunct in the treatment of abscesses without external fistulæ, as by its absorbent properties it draws the pus to itself, which is thus better removed than by any other process.

This frequent stopping and unstopping, using oil of cloves for its purifying and soothing effects, will often effect a cure in ten to twenty days.

When the tooth will bear a tight cotton stopping for eight or ten days, especially if there be no unpleasant odor on its withdrawal from the canal, it may be permanently filled.

If the abscess be situated at the end of an inaccessible root, or if after repeated trials it does not improve under the stopping treatment, the root may be tightly closed and a fistulous opening forced through the alveolar walls and gum. With judicious stimulation this may be accomplished in from two to four days, when the case is reduced to one of the simplest form, and should be treated as already described.

The stimulation in such cases may consist of an application, every ten to twenty minutes, of a mixture of equal parts of tincture of capsicum and chloroform (well shaken,) applied on a pellet of cotton over the surface where it is desired to establish the fistula. This, while an excellent stimulant, is difficult for the inexperienced to apply, for which cause it has been almost entirely supplanted, in my practice, by what are known as "pepper bags," a device of my friend and former colleague, Dr. J. Foster Flagg.

Pieces of thin muslin and of the lightest muslin rubber, about one half by three-fourths of an inch each, are sewed together on three sides, making an open sack.

Into this sack are introduced equal parts of powdered capsicum and ginger, covering the inner surface to a thickness equaling, perhaps, an ordinary blotting pad. The bag is then closed by sewing up the fourth side.

These little pepper-bags, dipped in water before using, are applied muslin side to the gum of the affected tooth; by this means constant and bearable stimulation can be kept up, as the medicament acts readily through the muslin; while the rubber of the other side being impervious, protects the mucous membrane of the lip or cheek. This device is as ingenious as it is useful.

The diagnosis of an abscess at the *bifurcation* of the root is often exceedingly difficult.

The diagnostic signs are, almost equal response to pressure in various directions; inability to use the tooth in mastication, and *movement* of the tooth, greater or less, in its socket. The liability to such form of abscesses is chiefly from anatomical formation in the lower molars.

The treatment consists in evacuation of the pus from the alveolar socket. This can sometimes be accomplished by trephining the alveolar plate into the bifurcation, but more commonly by drilling through the tooth from the pulp-cavity directly upon the abscess, and afterwards injecting it occasionally with dilute sulphuric acid (acid 1 part, water 3 parts.)

If a cure from medication is possible, it can generally be effected in this manner.

As a last resort, the tooth may be extracted, the abscess dissected from it (for the abscess will always come away with the tooth,) and the tooth replanted.

With the most skillful treatment, a considerable percentage of cases will result in failure and eventuate in extraction and loss.

An alveolo-dental abscess dissecting the peridentium from the cementum is generally associated with an anæmic, possibly a typhoid, condition, or with want of tonicity of the tissues generally. The marked local symptoms are sensitiveness to pressure, looseness of the tooth, and the oozing of pus from about its neck. The treatment of these cases is always difficult and often unsuccessful.

If the tooth be situated in the lower jaw, unless specially valuable, early extraction is the safest plan to pursue. In the effort to save the tooth, the pus should, if possible, be diverted from the channel which it has established for itself to an opening made through the *alveolus* and *gum*. If the abscess be then treated with dilute sulphuric acid, and the peridentium with applications of chloride of zinc, a satisfactory cure may generally be expected.

Abscesses with external fistulæ through the cheek are rare, but a good prognosis can generally be given. The method of treatment will suggest itself from that indicated for other conditions.

In the lower jaw external fistulæ are not infrequent, gravity assisting materially to produce such an untoward result. If the subject be a female, the unsightly scar, which is the usual result, ever remains a disfigurement.

The sacrifice of the tooth giving rise to the trouble is not a necessity, for in a moderate percentage of cases the abscess may be successfully treated through the root. The external scar left from such an abscess is usually much less disfiguring if a cure is effected with the tooth in place; but unless it be especially valuable, it is safer, after the healing of the abscess, to extract to prevent recurrence.

The pulp-cavity and root should be thoroughly cleansed and disinfected, and then dressed with one of the medicaments (glycerine, oil of cloves, a saturated solution of salicylic acid in oil of cloves, dilute sulphuric acid,) as already recommended. By this treatment, provided the root be an accessible one, healthy granulations may be induced, and the abscess healed from the bottom.

For an abscess in connection with an inferior dens sapientiæ, situated in the parts in the angle of the jaw, the extraction of the tooth is always to be resorted to if it be possible of accomplishment. Often in such cases the tooth is but partially erupted, or the crown much decayed. This, with the swollen condition of the parts, and the consequent inability to distend the mouth, may present insuperable obstacles to extraction.

Such cases demand the most active treatment, with a view of inducing a discharge of the pus through the gums, or at the worst an external fistula.

In addition to what has been already recommended, poultices may be applied externally, incisions made, or such other treatment adopted as the exigencies of the case may demand. The danger consists in pus finding its way under the strong cervical fascia, and thence to the thorax.

In extracting for alveolo-dental abscess the after-treatment is often of great importance.

Should the sac come away with the root, the trouble is practically at an end; if it does not, unless properly cared for, a recurrence of the abscess may be looked for.

A solid clot forming in the socket will effectually confine the pus, causing a speedy renewal of all the symptoms, intense pain, rapid swelling, etc.

The diagnosis and treatment, although plain, are frequently wholly misunderstood, causing much needless suffering to the patient.

The socket of an abscessed tooth, after extraction, may be soothed and kept free from a hard clot by syringing occasionally with tepid water, or a mixture of equal parts of warm water and laudanum; or if a more active detergent is indicated, perhaps the best is a wash composed of one part of alcohol to two parts of *phenol sodique.—Dental Cosmos.*

